# Breast cancer survival and its prognostic factors in the United Arab Emirates: A retrospective study

**DOI:** 10.1371/journal.pone.0251118

**Published:** 2021-05-05

**Authors:** Yusra Elobaid, Maria Aamir, Michal Grivna, Abubaker Suliman, Samir Attoub, Hussam Mousa, Luai A. Ahmed, Abderrahim Oulhaj

**Affiliations:** 1 Department of Medical and Health Sciences, Khawarizmi International College, Abu Dhabi, United Arab Emirates; 2 Cancer Registry, Tawam Hospital, SEHA, Al Ain, United Arab Emirates; 3 Institute of Public Health, College of Medicine and Health Sciences, United Arab Emirates University, Al Ain, United Arab Emirates; 4 Department of Pharmacology and Therapeutics, College of Medicine and Health Sciences, United Arab Emirates University, Al-Ain, United Arab Emirates; 5 Department of Surgery, College of Medicine and Health Sciences, United Arab Emirates University, Al Ain, United Arab Emirates; 6 Zayed Center for Health Sciences, United Arab Emirates University, Al Ain, United Arab Emirates; Fondazione IRCCS Istituto Nazionale dei Tumori, ITALY

## Abstract

**Background:**

Data on breast cancer survival and its prognostic factors are lacking in the United Arab Emirates (UAE). Sociodemographic and pathologic factors have been studied widely in western populations but are very limited in this region. This study is the first to report breast cancer survival and investigate prognostic factors associated with its survival in the UAE.

**Methods:**

This is a retrospective cohort study involving 988 patients who were diagnosed and histologically confirmed with breast cancer between January 2008 and December 2012 at Tawam hospital, Al Ain, UAE. Patient were followed from the date of initial diagnosis until the date of death from any cause, lost-to-follow up or the end of December 2018. The primary outcome is overall survival (OS). The Kaplan-Meier method was used to estimate the survival curve along with the 2- and 5-year survivals. Different group of patients categorized according to prognostic factors were compared using the log-rank test. Multiple Cox proportional hazards models was used to examine the impact of several prognostic factors on the overall survival.

**Results:**

The median study follow-up was 35 months. Of the 988 patients, 62 had died during their follow-up, 56 were lost to follow-up and 870 were still alive at the end of the study. The average age of patients was 48 years. The majority of patients presented to the hospital with grade II or III, 24% with at least stage 3 and 9.2% had metastasis. The 2-year and 5-year survivals were estimated to 97% and 89% respectively. Results of the multiple Cox proportional hazard model show that tumor grade, and stage of cancer at presentation are jointly significantly associated with survival.

**Conclusion:**

The 2- and 5-year survival are within the norms compared to other countries. Significant clinical and pathological prognostic factors associated with survival were tumor grade, and the stage of cancer at presentation.

## Introduction

Breast cancer is a significant global public health concern, since it is the most common cancer among women [1]. Survival rates differ across the globe with higher survival rates in developed compared to less developed countries. For instance, the 5-year survival rate in developed countries such as USA and UK was in the range of 85–90% between the years 2017–2019 [2, 3]. In developing countries this rate ranged between 40–60% [1, 4, 5]. The poorer survival in developing countries could be related to low awareness of screening needs, lack of early detection programs and lack of diagnosis and treatment facilities [6].

Many improvements have been made in the last 20 years in the management of breast cancer due to the identification of prognostic factors capable of providing information on the progression of the disease. These prognostic markers are usually indicators of growth, invasion, and metastatic potential [7]. These include tumor size, lymph nodes, histologic grade, stage, estrogen and progesterone receptors and HER2/neu (erB-2) oncogene alteration [8].

In the United Arab Emirates (UAE), breast cancer constitutes approximately 20% of all cases of cancer and is considered to be the second leading cause of death among women after cardiovascular diseases [9]. Although, the burden of breast cancer is not systematically investigated in the country, the available information indicates that the cumulative probability of developing breast cancer in the UAE increased over the past three decades, being 2% in 1980, 2.4% in 1990, 3.9% in 2000 and 5.2% in 2010 [10]. Although this rise in cumulative probability could be explained by the improvement in screening program, this rising incidence is not the only cause of concern in the UAE, but also the advanced stage at presentation [11]. Metastasis is also common among women in the UAE where around 15% of cases are metastatic [9].

Despite few studies have been published in the UAE on breast cancer research, there is still a gap of knowledge in this field. There is very limited data in the UAE and the region on the survival rates and prognostic factors for breast cancer. To the best of our knowledge, no study has been yet carried out in the UAE to estimate the survival rates of breast cancer. The aim of this study is to estimate the survival curve along with the 2-year and 5-year survival in breast cancer patients in the UAE, and also to evaluate the impact of multiple prognostic factors on the breast cancer survival. Knowledge of these prognostic factors in the UAE population will be the foundation for planning treatment and predicting the outcome for patients with breast cancer.

## Methods

### Population and study design

This is a retrospective cohort study involving all patients (n = 988) who were diagnosed (and histologically confirmed) with breast cancer between January 2008 and December 2012 at the department of oncology at Tawam hospital, a tertiary care and teaching hospital located in Al Ain (Eastern region of the Emirate of Abu Dhabi). Each patient was followed up from the date of initial diagnosis until the date of death from any cause or until 31^st^ December, 2018. Data collected at baseline (date of diagnosis) included demographic variables (such as age and sex), anatomical site and quadrant (primary site), tumor size, pathological nodal status, clinical stage, histopathological type and grade and treatment modalities. The lymph node and tumor size status were pathologically evaluated. Outcome data (at follow-up) included date of death if occurred or date of last contact with the patient. This information was collected using a pre-formed questionnaire. Ethical approval to conduct the study was obtained from the Al Ain District Human Ethics Committee before the commencement of the study.

#### Determination of histological type, grade and stage using TNM classification

Determination of histological type and grade was established by Modified Bloom-Richardson score system which scores for tubular formation, nuclear pleomorphic and mitotic rate within tumor cells. TNM was used to assign the clinical stage of the disease to each patient [12]. This is a staging system which is expression of anatomical extent of disease based on extent of primary tumor (T), absence or presence of and extent of regional lymph node metastasis (N) and absence or presence of distant metastasis (M).

### Statistical analysis

Baseline characteristics were summarized using descriptive statistics including mean, median, and standard deviation for continuous measures, and frequencies tables for categorical variables. Categorical variables were compared using the chi square or Fisher’s exact test and continuous variables using the unpaired t-test. The primary outcome is overall survival (OS). The survival time was defined as the duration of time from the date of initial diagnosis until the date of death from any cause, date lost-to-follow up or the end of December 2018. Survival curves were estimated and plotted using the Kaplan-Meier method. Survival curves of different groups were compared using the log-rank test. Cox proportional hazards models were applied to examine the impact of prognostic factors on overall survival. These included the age at diagnosis, treatment delay (duration of time from the date of initial diagnosis to the date of starting the treatment), tumor grade, stage of cancer, metastasis, primary site and laterality. Pairwise associations between all these prognostic factors was first carried out, using the Goodman and Kruskal’s tau [13], in order to identify factors providing redundant or similar information on overall survival. This is done to avoid issues of multi-collinearity when fitting Cox proportional hazards models. Lasso procedure was used as a selection method to identify the set of risk factors that are jointly significantly associated with overall survival. The 2-year and 5-year chance of survival were derived from the fitted model. The results of Cox proportional hazard model are presented as hazard ratios along with their 95% confidence intervals. All statistical tests were two-sided, and p-values < .05 were considered statistically significant. All the analysis was conducted using R software version 3.6 [14].

## Results

### Baseline characteristics of the patients

The final data set includes 988 breast cancer female patients having complete information on their demographic, prognosis factors and follow-up duration. Of these 988 patients, 62 had died during the follow-up, 56 were lost to follow-up and 870 were still alive at their last follow-up. The maximum follow-up time was 125 months and the median follow-up was of 35 months.

The average age at diagnosis was 48 years. The distribution of demographics, clinical and pathological characteristics at baseline, overall and according to the age group of diagnosis (< 50 years versus > 50 years) are presented in Table 1. The majority of patients are non-Emirati (81%). The average duration of time between the date of initial diagnosis and the date of first treatment was 18 days.

**Table 1:** Demographic, clinical and pathological characteristics at baseline, overall and by age groups.

Variables	Total (n = 988)	< 50 years (n = 570)	≥ 50 years (n = 418)	P-value†
Age in years, Mean (±SD)	48.4 (±11.7)	40.4 (±6.3)	59.2 (±8.0)	<0.001
Delay in treatment (days), Mean (±SD)	27.4 (±37.9)	29.3 (±42.4)	24.7 (±30.6)	0.198
Delay in treatment (days) by median, n (%)				
≤18 days	494 (50.0%)	278 (48.8%)	216 (51.7%)	0.403
>18 days	494 (50.0%)	292 (51.2%)	202 (48.3%)	
Grade, n (%)				
Grade I	117 (11.8%)	63 (11.1%)	54 (12.9%)	0.204
Grade II	392 (39.7%)	217 (38.1%)	175 (41.9%)	
Grade III	479 (48.5%)	290 (50.9%)	189 (45.2%)	
Laterality, n (%)				
Left	533 (53.9%)	286 (50.2%)	247 (59.1%)	0.007*
Right	455 (46.1%)	284 (49.8%)	171 (40.9%)	
Tumor Size, n (%)				
T0,Tis,T1,T2	755 (76.4%)	430 (75.4%)	325 (77.8%)	0.404
T3,T4	80 (8.1%)	50 (8.8%)	30 (7.2%)	
Missing**	153 (15.5%)	90 (15.8%)	63 (15.1%)	
Lymph Node status, n (%)				
N0,N1mi	314 (31.8%)	180 (31.6%)	134 (32.1%)	0.942
N1,N2	521 (52.7%)	300 (52.6%)	221 (52.9%)	
N3	62 (6.3%)	37 (6.5%)	25 (6.0%)	
Missing**	91 (9.2%)	53 (9.3%)	38 (9.1%)	
Metastasis, n (%)				
M0	897 (90.8%)	517 (90.7%)	380 (90.9%)	1.000
M1	91 (9.2%)	53 (9.3%)	38 (9.1%)	
Stage, n (%)				
0, 1	200 (20.2%)	115 (20.2%)	85 (20.3%)	0.837
2	452 (45.7%)	255 (44.7%)	197 (47.1%)	
3	245 (24.8%)	147 (25.8%)	98 (23.4%)	
4	91 (9.2%)	53 (9.3%)	38 (9.1%)	

† Continuous variables were summarized using the T-test, while categorical variables were summarized using Pearson’s Chi-squared test.

* Significant association (P-value < 5%).

** Tumor size was not evaluated for patients with tumor stage IIIC or IV. Lymph node was not evaluated for patients with tumor stage IV.

The majority of patients (88%) were presented with grade II or III and approximately 54% had left laterality. Regarding the stage of breast cancer, 80% of the patients were diagnosed with stage 2 at least. There was no statistically significant association between the tumor grade and the age at diagnosis. The distribution of tumor grades was similar in younger (< 50 years) compared to older woman (> 50 years) (P-value = 0.204). The same conclusions apply to other prognostic factors such as metastasis and stage of cancer except for laterality where older women were found to be more likely to have breast cancer in the left side compared to younger women.

#### Survival curves

**Fig 1:**
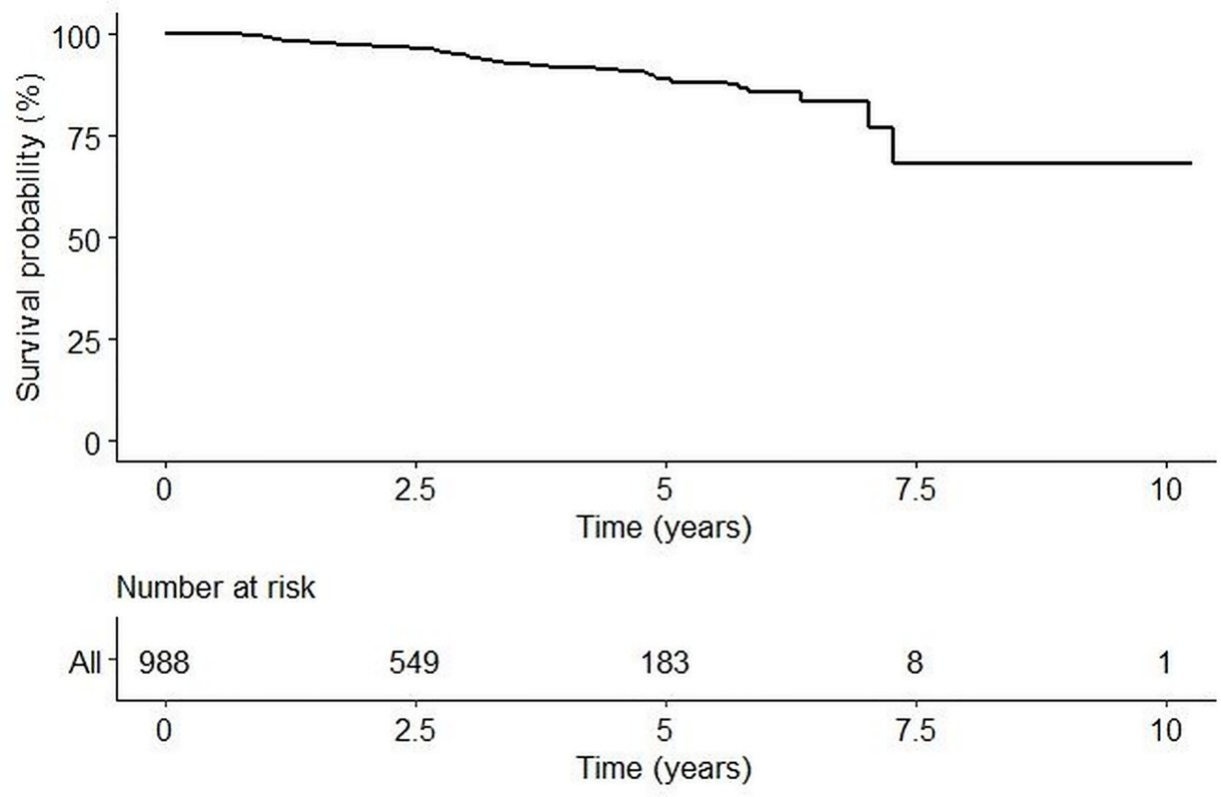
Overall survival curve.

The unadjusted survival curve of patients, estimated using Kaplan-Meier method, is illustrated in Fig 1. Unadjusted survival curve. The 2-years survival was 97% (95% CI; 96%-99%) meaning that 97% of breast cancer women survived beyond 2 the first two years after their initial diagnosis and the 5-years survival was estimated to 89% (95% CI; 86%-92%).

**Fig 2:**
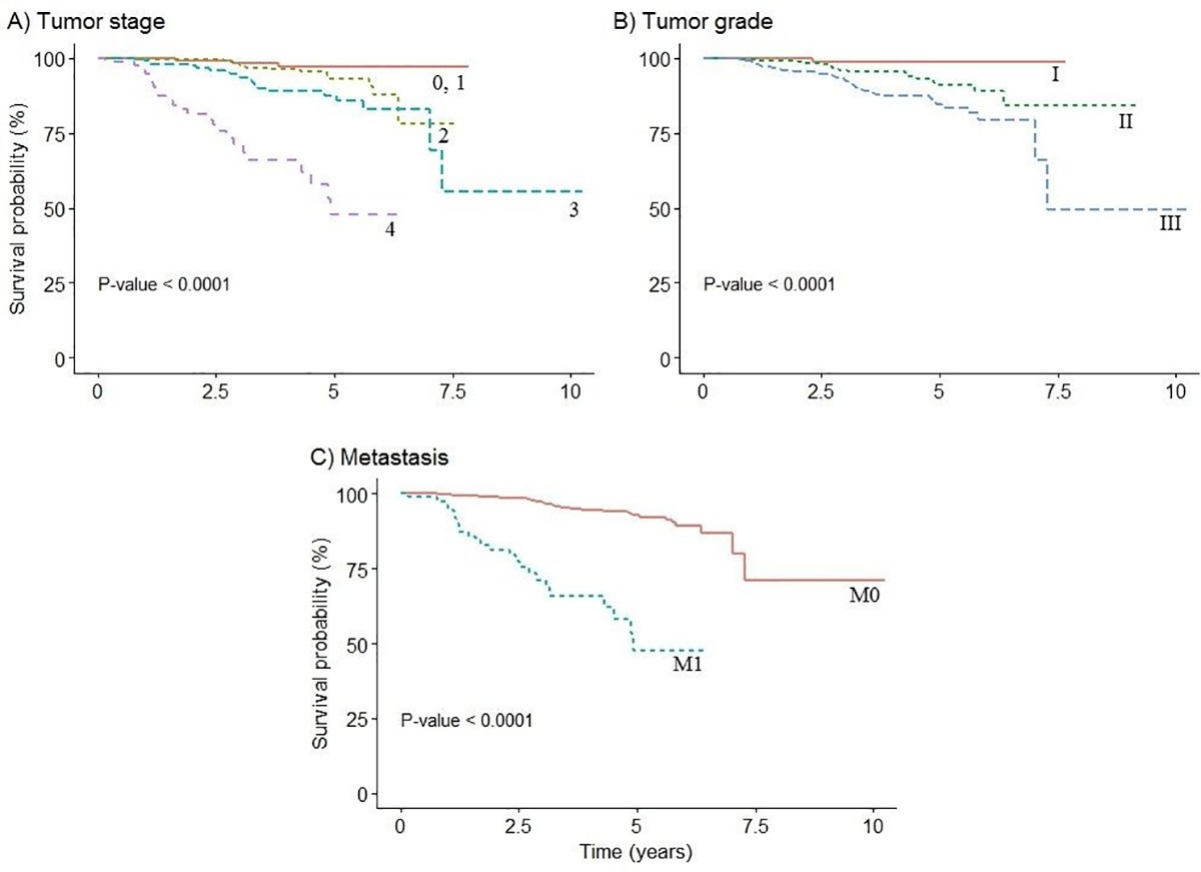
Overall survival curve according to selected prognostic factors.

Fig 2 shows the estimated survival functions according to the stage of cancer, tumor grade and metastasis. The log-rank test shows significant differences in survival curves between groups of each prognosis factor (all p-values were < 0.001). Patients with tumor grade I were observed to have higher 5-years survival of 99% (95% CI; 96%-100%) compared to those with tumor grade III who had a low 5-years survival rate of 85% (95% CI; 80%-90%). Similarly, patients with M0 metastasis had higher 5-years survival of 93% (95% CI; 90%-95%) compared to patients with M1 metastasis who had a 5-years survival of 48% (95% CI; 33%-70%). Regarding the stage of breast cancer, it is clear that those diagnosed with advanced stage have worse survival diagnosis 48% (95% CI; 33%-70%) in comparison to those with stage 1 or less 97% (95% CI; 94%-100%).

#### Cox proportional hazard model

The hazard ratios obtained from separate univariate cox proportional hazard models fitted for each potential prognosis factor show no evidence for the survival curve to be affected by the age at diagnosis or the relative delay in treatment (in months). However, among clinical and pathological factors investigated, only the tumor grade and stage of cancer were statistically significantly associated with the survival (Table 2).

**Table 2:** Crude hazard ratio of overall survival associated with several prognostic factors.

Variables	Value	Hazard ratio (95% CI)	P-value
Age, Mean (±SD)	48.4 (±11.6)	1.01 (0.99–1.03)	0.502
Age by category, n (%)			
≤35 years	122 (12.3%)	1	0.516
36–49 years	449 (45.4%)	0.81 (0.38–1.74)	
50–64 years	326 (32.9%)	0.74 (0.33–1.63)	
≥65 years	93 (9.4%)	1.38 (0.53–3.59)	
Delay in treatment (days), Median (range)	18.0 (0.0–443.0)	1.00 (0.99–1.00)	0.415
Delay treatment by Median (days), n (%)			
≤18	496 (50.1%)	1	0.340
>18	494 (49.9%)	0.78 (0.48–1.29)	
Grade, n (%)			
Grade I	117 (11.8%)	1	< 0.001
Grade II	392 (39.6%)	5.86 (0.78–43.90)	
Grade III	481 (48.6%)	12.35 (1.70–89.70)	
Laterality, n (%)			
Left	534 (53.9%)	1	0.077
Right	456 (46.1%)	0.63 (0.37–1.06)	
Stage, n (%)			
0, 1	200 (20.2%)	1	< 0.001
2	454 (45.9%)	2.76 (0.80–9.54)	
3	245 (24.7%)	5.92 (1.76–19.93)	
4	91 (9.2%)	29.09 (8.72–97.02)	
Metastasis, n (%)			
M0	899 (90.8%)	1	< 0.001
M1	91 (9.2%)	9.12 (5.43–15.33)	

The final multiple Cox proportional hazard model, derived from the stepwise backward and the lasso variable selection procedures, retained two prognosis factors that are jointly significantly associated with survival. These include tumor grade and the stage of cancer. The results of the fit of the final Cox proportional hazard model, along with the estimated hazard ratio and their corresponding 95% confidence intervals are provided in Table 3.

**Table 3:** Results of final multivariate of the Cox proportional hazard model.

	Hazard ratio (95% CI)	P-value
Tumor grade		
Grade I	Ref	-
Grade II	4.90 (0.65–36.88)	0.122
Grade III	10.24 (1.40–74.89)	0.022*
Stage		
0, 1	Ref	-
2	2.41 (0.69–8.34)	0.166
3	4.85 (1.44–16.36)	0.011
4	25.76 (7.70–86.21)	<0.001

* Significant association (P-value < 5%).

The hazard ratio of death in patients with tumor grade III was 10.24 compared to those with Grade I. Patients presented at earlier stage were more likely to survive compared to those presented at late stage. More specifically, the hazard ratio was 4.85 for patients with stage 3 and 25.76 in patients with stage 4 compared to those with stage I.

## Discussion

Clinical and histological information of a large number of breast cancer patients recruited from a tertiary hospital-based cancer registry in the UAE were analyzed. The aim of the study is to estimate the overall survival curve and the 2- and 5-year survival in breast cancer patients, and also to identify the set of important prognostic factors affecting survival in these patients. The average age of diagnosis was 48 years with the majority of patients presenting to the hospital with grade II or grade III, 24% with at least stage 3, and 9.2% had metastasis. We showed that among the clinical and pathological risk factors investigated, the tumor grade and the stage of cancer at presentation and metastasis were statistically significantly associated with the overall survival when analyzed separately. However, when added together into the Cox proportional hazard model, only tumor grade and stage of cancer were found to be jointly significantly associated with overall survival. The 5-year risk of death from breast cancer was estimated to 11% and the 2-year risk to 3%.

Based on the CONCORD 3 study [15], in the gulf region, breast cancer survival rates increased between 1995–1999 and 2010–2014. In Saudi Arabia, between 1995–1999 and 2005–2009 the overall 5- year’s survival increased from 70.9% to 78.4%. In Bahrain, between 2000 and 2004 the survival was reported to be 63%. In Kuwait, between 2000–2004 and 2010–2014, the survival increased from 68.3% to 75.2% and in Qatar, it has increased from 59.2% to 71.9%. In the UAE, there is no previously reported disease survival rate, this is the first study to report it. Based on our findings, the 5 years survival of breast cancer in the UAE was 89%, this is similar to western countries such as Australia 89.5% and Canada 88.2% [15]. The 5 year survival rate is also good compared to other countries in the same region such as Qatar (71.95%) and Kuwait (75.2%). This may reflect improved treatment and intensified efforts to tackle the burden of breast cancer in the country through the introduction of the national screening program. Previous studies have established numerous prognostic factors of breast cancer survival including age at diagnosis, tumor size, axillary lymph node involvement, tumor grade, stage of cancer, and metastasis. In our study, the average age at diagnosis of women was 48 years supporting the findings of other studies from the region claiming that Arab women are more likely to develop breast cancer at an earlier age [16, 17]. In fact, the median age at diagnosis of breast cancer in the UAE is 10 to 15 years younger than in North America and Europe [18]. Women at younger ages generally suffer from a more aggressive type of breast cancer, an advanced stage at presentation, and worse outcome [19]. A study conducted in southern Iran showed a relatively low 5-year survival (58%) of breast cancer [20]. However, in our data set, the hazard ratios obtained from separate univariate cox proportional hazard models fitted for each variable show no evidence for the survival function to be affected by age at diagnosis. In this study, the median patient delay in seeking treatment was 18 days, this is relatively similar to other studies in Europe [21, 22]. The percentage of stage 1 and II breast cancer in Australia, Canada, Denmark, Norway, Sweden, and the United Kingdom was 61–62% between 2000–2007 [23]. In our study, around 66% of patients were diagnosed at stage I or II versus 34% in stage III or IV. This was comparable to data from developed countries.

In this study, tumor stage was related to decreased survival, similar to other researches [24–26]. Saadatmand et al (2015) reported that in univariable and multivariable analyses, both stage and lymph node status had major influence on overall survival. Stage at diagnosis still influence overall survival considerably [27].

In our study, the hazard ratio for death in patients with grade III compared to those with Grade I was 10.59 (95% CI 1.45–77.44). This finding is in agreement with the literature, suggesting that more advanced grading has worse prognoses for breast cancer [28, 29].

This is the first study to report estimates of survival rates and prognostics factors of breast cancer based on cancer registry data in the UAE. The study was based on robust cancer registry data. The follow-up duration was sufficient to capture and adequately estimate the 2-year and 5-year risk of death due to the fact that 50% of subjects were followed up for more than 35 months and 25% of patients followed up for more than 56 months. However, as this was hospital-based registry, generalizability of the study findings should be interpreted with caution. The sample does not represent the whole UAE, but it captures a good proportion of breast cancer patients referred to Tawam Hospital as it is used to be the main referral center for oncology cases in the UAE and has the only breast health center in the country. Moreover, as the analysis was based on secondary data, other potential confounding factors such as presenting symptoms and signs, family history, and lifestyle factors including diet and physical activity were unavailable and could not be included in the study analysis.

## Conclusion

The 2-year survival was estimated to 97% and the 5-year survival to 89%. Tumor grade and the stage of cancer were found to be jointly significantly associated with survival. The findings of this research enable the estimate of the long-term breast cancer survival, as well as reemphasizes the importance of early diagnosis and screening through mammography which can prevent local systemic metastasis and therefore advanced stage diagnosis.
